# Supplementary material for: Electrochemical Characterization of Two Gut Microbial Strains Cooperatively Promoting Multiple Sclerosis Pathogenesis
Source: Microorganisms. 2024 Jan 25;12(2):257. doi: 10.3390/microorganisms12020257 (PMC10892914; doi:10.3390/microorganisms12020257)
Supplement: Supplementary file 1 [file microorganisms-12-00257-s001.zip › microorganisms-2812592-supplementary.pdf]

Supplementary Figures

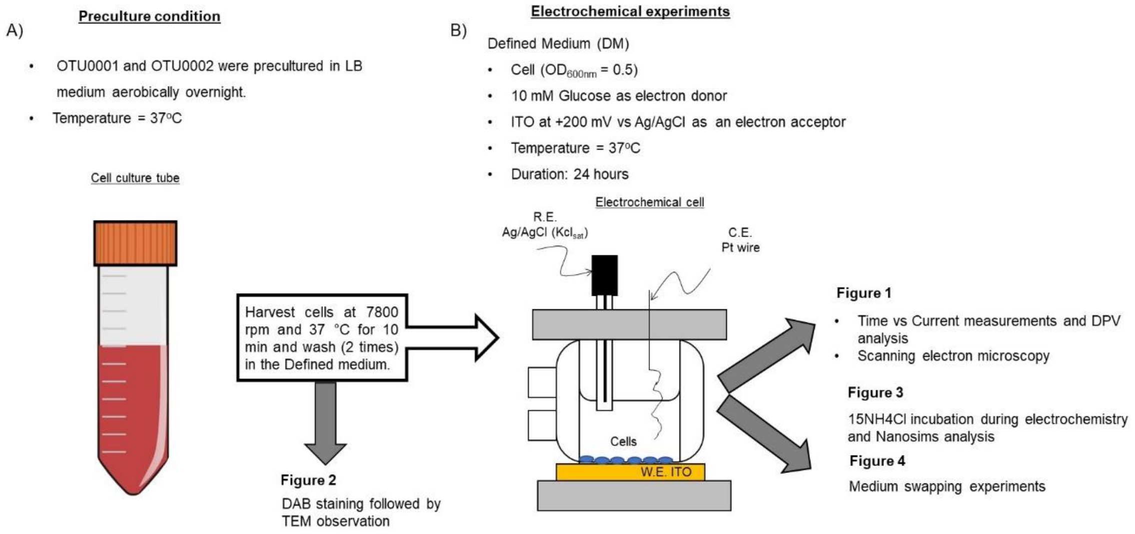

Figure S1. An illustration showing the experimental procedures that were used in this study **(A)** Preculture growth medium and bottle condition used for OTU0001 and OTU0002 growth, which were harvested, washed in defined medium (DM), and used for electrochemical experiments. Similar cells were subjected to 3,3'-diaminobenzidine (DAB) staining followed by TEM observations as presented in Figure 2. **(B)** Electrochemical setup containing three electrodes, indium tin-doped oxide (ITO) electrode (surface area: 3.14 cm^2) poised at +0.4 V (versus SHE) as working electrode (W.E.), platinum wire as counter electrode (C.E.) and Ag/AgCl (saturated KCL) as reference electrode (R.E.). Separate electrochemical setups were used to obtain the Figures 1, 3, and Figure 4 data. More than two repeated experimental analyses were conducted and representative data was shown.

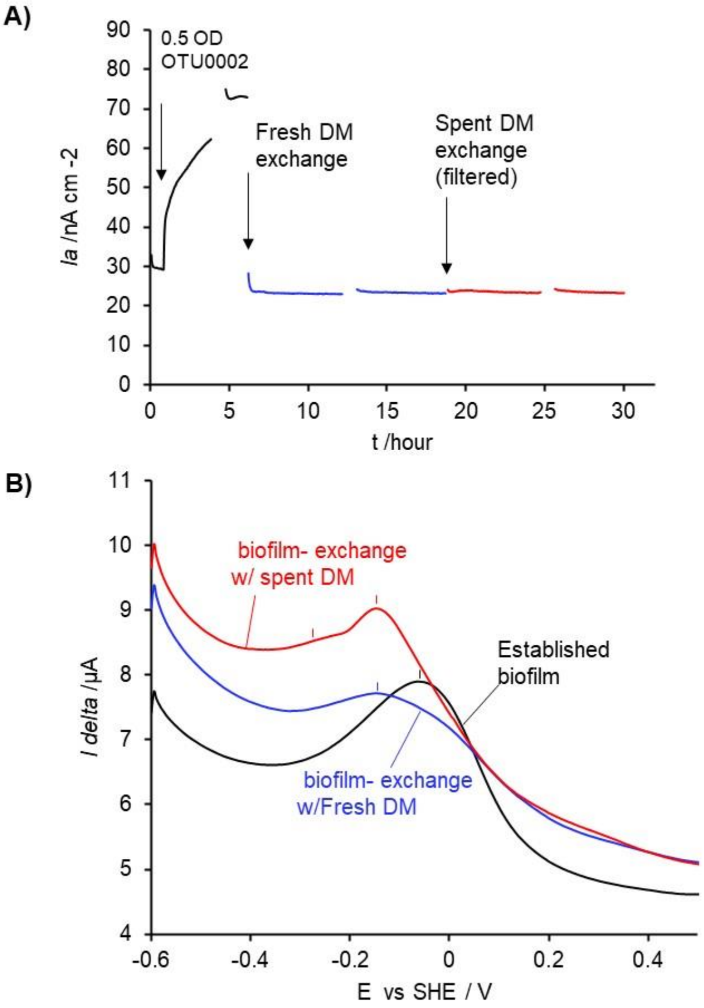

Figure S2. Medium swapping analysis of OTU0002 in the electrochemical reactor. A) Supernatant replacement during the current production of OTU0002 at +0.4 V (versus SHE). At the indicated times, the medium was removed and replaced with a fresh defined medium containing 10 mM glucose (blue line) or cell-free spent medium (red line), leading to a decrease in current production. The same tendency was confirmed in two individual experiments. B) Differential pulse voltammograms in the presence of OTU0002 before (black line), after fresh medium exchange (blue line), and DPV of biofilm after spent medium exchange (red dotted line).

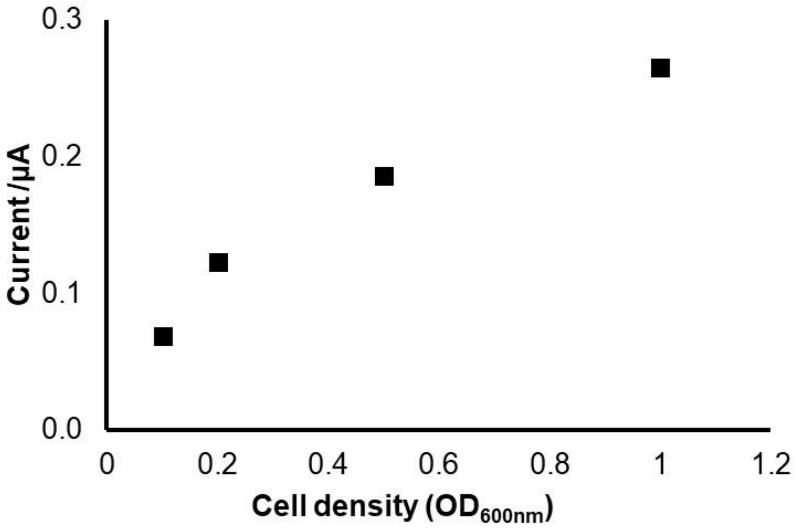

Figure S3. Correlation between cell density (OD_{600nm}) and maximum current produced (µA) by OTU0002 taken from Figure 2A Time vs Current profile of different cell densities 0.1, 0.2, 0.5, and 1.0, showing the non-linearity.

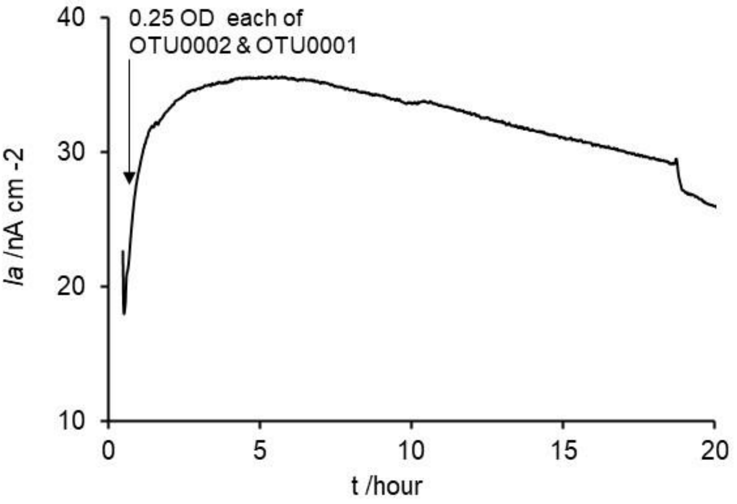

Figure S4. Current vs time measurements of mixed culture of OTU0001 and OTU0002, conducted in anaerobic reactors equipped with ITO electrodes (surface area: 3.14 cm^2) poised at +0.2 V vs Ag/AgCl in the presence of 10 mM glucose and the OD_{600} of 0.25 of each strain initial cell density added to the reactors. The arrow position indicates the time of cell addition in the electrochemical reactor.

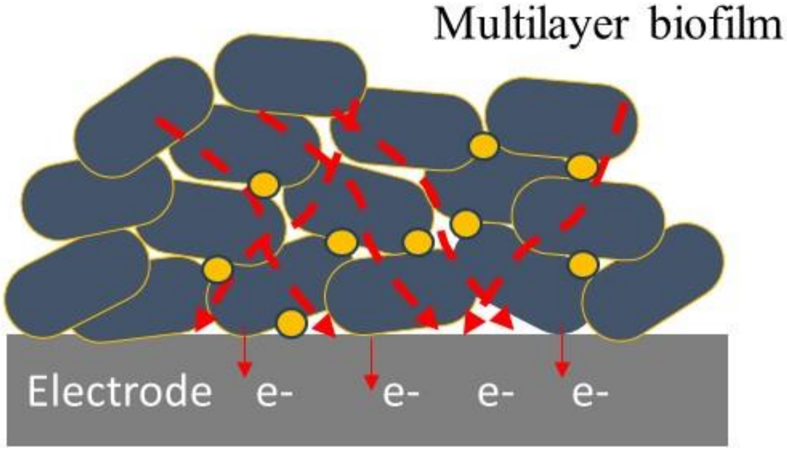

Figure S5. Schematic of possible long-range electron transport mechanism by OTU0002 cells. At high OD_{600} the EET is enhanced by the concentration of cell-released redox active product, which acts as an electron mediator. The cell-released mediator attaches to the cell and transfers the electrons to the electrode via reduction and oxidation state cycling.
